# Gray matter heterotopia: clinical and neuroimaging report on 22 children

**DOI:** 10.1007/s13760-021-01774-3

**Published:** 2021-09-01

**Authors:** A. Di Nora, G. Costanza, F. Pizzo, C. F. Oliva, A. Di Mari, F. Greco, P. Pavone

**Affiliations:** 1 Department of Clinical and Experimental Medicine, University of Catania, Via S. Sofia 78, 95123 Catania, Italy; 2 Department of Radiology, University of Catania, Catania, Italy; 3 Department of Clinical and Experimental Medicine, Section of Pediatrics and Child Neuropsychiatry, Hospital “Policlinico G. Rodolico”, Catania, Italy

**Keywords:** Heterotopia, Childhood, Clinical, Neuroimaging, Epilepsy

## Abstract

**Objective:**

To investigate the clinical characteristics and neuroimaging features of childhood presenting with gray matter heterotopia observed in a single tertiary Pediatric Department in Catania and compare the data with those reported in the literature.

**Methods:**

A retrospectively review of the history, clinical findings, electrophysiological features and magnetic resonance images of 22 children presenting with gray matter heterotopia observed from January 2010 to January 2020.

**Results:**

Among the 22 children included in the study, 17 presented with periventricular heterotopia (PVNH), two with Subcortical Band Heterotopia (SBH), and three with other subcortical heterotopia (SUBH). In the affected children, the ages at first diagnosis ranged from 3 months to 16 years with a mean age of 8.2 years (± 5.4); twelve (54.5%) suffered by developmental delay and intellectual deficit; eleven children (50%) complained of epileptic seizures, mostly focal to bilateral tonic–clonic seizure. In addition, in the periventricular heterotopia group (PVNH), cerebral and systemic malformations were reported in twelve (70%) and in ten (58%) children, respectively, out of seventeen. In the SBH plus SUBH group, epileptic seizures were recorded in 3 (60%) out of 5 children, cerebral malformations in one child and systemic malformations in two children.

**Conclusions:**

Heterotopic gray matter malformations include a group of disorders that manifest with a variety of neurological implications, such as cognitive impairment and epilepsy, and often related with epilepsy, other cerebral malformations and systemic anomalies.

## Introduction

Heterotopic gray matter malformations (HET) are clusters of normal neurons in abnormal locations, mainly due to impaired migration from approximately the 6th to 16th weeks of gestation [1–3]. The increasing availability and resolution of MRI technology and molecular genetics has resulted in a classification published by Barkovich et al. in the 2012 describing different types of malformations of cortical development (MCD) [4]. In 2019, Oegema et al. reviewed this classification [5]. In particular, he distinguished five groups and subdivided them into specific entities. Subcortical Heterotopic gray matter heterotopy (SUBH) was included in Groups 1, 2, 4 and 5, differently periventricular nodular heterotopia (PVNH) and Subcortical Band Heterotopia (SBH) in group 3. In particular, Group 1 includes congenital microcephaly with premigrational reduced proliferation and variable additional MCD; group 2 includes multifocal or focal cortical and subcortical dysgenesis; group 3 includes malformations due to abnormal neuronal migration, PVNH and SBH [6–10]; group 4 includes malformations due to abnormal postmigrational development with subcortical or transmantle components; and group 5 includes heterotopic gray matter brain malformations with bilateral complex patterns.

The diagnosis of GMH requires highly specialized and multidisciplinary expertise. Clinically, it has been commonly related to developmental delay and other systemic malformations. The purpose of this study was to evaluate the clinical and neuroimaging features of gray matter heterotopia observed over a 10-year period in a single tertiary pediatric department to improve the clinicians’ and radiologists’ understanding of the disease.

## Methods

We reviewed retrospectively the clinical records of the 22 children presenting with epilepsy and/or development delay and diagnosed at Brain MRI with HET, admitted at single tertiary Pediatric Department at the “Policlinico G. Rodolico” University-Hospital, Catania, Italy from January 2010 to January 2020. In this study, children were grouped according to the main types of heterotopia: seventeen had PVNH (77%), three SBH (13%), and two SUBH (9%). The study was approved by the ethic Committee of the University Hospital of Catania University. We collected the data from the medical charts: sex, age at first diagnosis, seizure types, electroencephalograpic (EEG) results, neuropsychiatric evaluation, were reported together with other possible cerebral or systemic malformations presented by the children. Brain MRI studies included T1-weighted, T2-weighted, fluid-attenuated inversion recovery, diffusion-weighted and post-contrast T1 imaging. EEG was carried out for each child using the international 10–20 system.

## Results

The age at first diagnosis ranged from 3 months to 16 years with a mean age of 8.2 years (± 5.4). Twelve children were males (54.6%) and ten (45.4%) were females. The results obtained are distinguished on the basis of the type of HET and beneath reported:

### Periventricular nodular heterotopia (PVNH)

This group of children includes nine females (52.9%) and eight males (47.1%), with mean age of establishing a diagnosis of 8.1 years (± 6.1). This group of children includes nine females (52%) and eight males (47%), with mean age of the first diagnosis of 8 years (± 6).

**Fig. 1:**
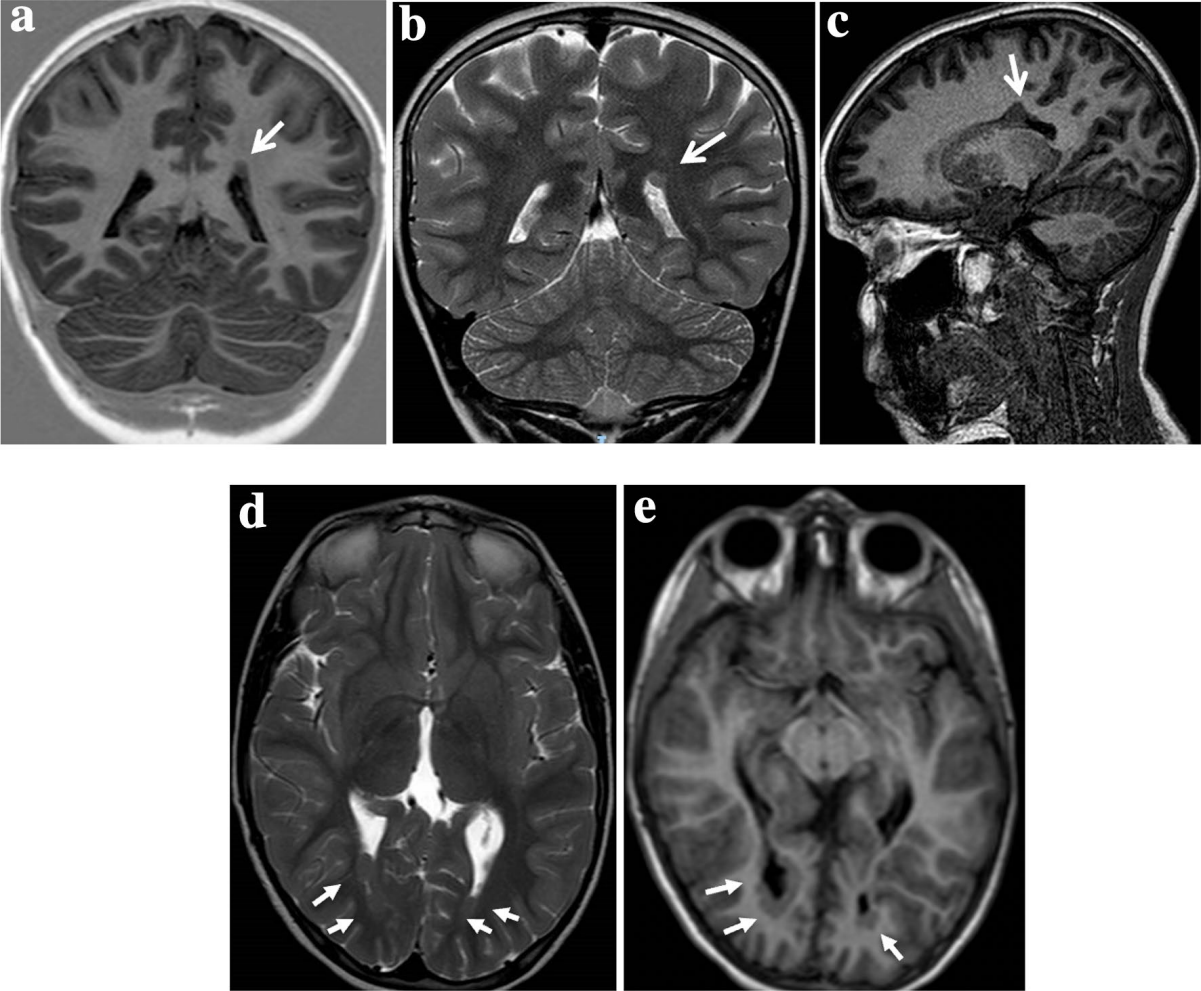
*Periventricular nodular heterotopia (PVNH)*. **a**, **b**, **c** *Unilateral PVNH (ID 101)*: Unenhanced magnetic resonance imaging coronal **a** and mid sagittal **c** T1-weighted and coronal T2-weighted **b** shows a small unilateral periventricular nodule, isointense to the gray matter, along the left lateral ventricular wall (arrows) with indentation of the ventricular profile (arrow). **d**, **e** *Bilateral PVNH (ID103)* Unenhanced T2-weighted **d** and T1-weighted **e** magnetic resonance axial images shows a few nodules that are lining ventricular occipital horns bilaterally (arrows)

Table 1 shows the clinical features. Figure 1 shows the main characteristics and intensity of periventricular nodules on MRI.

**Table 1:** Data of 17 patients with periventricular heterotopia

ID patient	Patient sex age at the diagnosis	Seizure	Seizure types	EEG	Heterotopic localisation	Outcome	Other CNS malformations	Systemic malformations/manifestations
ID-101	Female 10.75 y	Yes	FS	Left FE	Unilateral	Delayed	No	No
ID-102	Male 13.3 y	Yes	F to Bil	Right FE	Unilateral	Normal	No	No
ID-103	Female 8.3 y	No		Normal	Bilateral	Delayed	VM Hemispheric cyst AVM PVL Polymicrogyria CVH	Skeletal Dysplasia Ostium secundum atrial septal defect
ID-104	Female 0.25 y	Yes	IS	Multifocal FE	Unilateral	Normal	ACC Aicardi Syndrome	No
ID-105	Female 0.75 y	Yes	IS	GE	Bilateral	Normal	VM	Congenital Hypertrophic cardiomyopathy
ID-106	Male 4.25 y	Yes	GS	Multifocal FE	Bilateral	Delayed	VM Microcephaly Polymicrogyria	No
ID-107	Male 3 y	Yes	GS	Normal	Unilateral	Delayed	No	Classical Ehlerse–Danlos syndrome (COL5A1)
ID-108	Female 1.6 y	No		Normal	Bilateral	Normal	VM ACC CVH Dandye-Walker cyst	Strabismus
ID-109	Male 2 y	No		Normal	Bilateral	Delayed	VM	Skeletal dysplasia Strabismus
ID-110	Female 0.8 y	No		Normal	Unilateral	Delayed	No	No
ID-111	Female 15 y	Yes	F to bil	Right FE	Bilateral	Normal	No	GH deficiency
ID-112	Male 12.3 y	No		Normal	Unilateral	Delayed	VM ACC	Hypertelorism Nystagmus Conjunctival hypertrophy
ID-113	Male 14 y	No		Normal	Bilateral	Delayed	VM	Papillary edema
ID-114	Male 2 y	No		Normal	Bilateral	Normal	Hemispheric cyst	Cortical cysts in the right kidney
ID-115	Female 12 y	No		Normal	Unilateral	Enuresis Encopresis	Spinal Dysraphism with meningocele and tethered cord	No
ID-116	Female 16.4 y	Yes	FS	GE	Unilateral	Normal	VM	Bicuspid aortic valve GH deficiency
ID-117	Male 15 y	No		Normal	Bilateral	Delayed	Ectopic neurohypophysis	No

*ACC* agenesis of corpus callosum, *AVM* arteriovenous malformation, *CHD* congenital heart disease, *CVH* cerebellar vermis hypoplasia, *FE* focal epileptiform, *FS* focal seizure, *F to Bil* Focal to Bilateral tonic–clonic seizures, *Y* years, *PVL* periventricular leukomalacia, *VM* ventriculomegaly, *GE* generalized epileptiform, *GH* growth hormone, *GS* generalized seizure, *IBD* inflammatory Bowel Disease, *IS* infantile spasms

### Subcortical band heterotopia (SBH)

This group includes two children, one female and one male, with mean age of establishing a diagnosis of 7.5 years (± 5.5). Table 2 shows clinical features.

**Table 2:** Data of two children with SBH

ID patient	Patient sex age at the diagnosis	Seizure	Seizure types	EEG	Heterotopic Localisation	Psychomotor development	Other CNS malformations	Systemic malformations/manifestations
ID-201	Female 11.3 y	No		Normal	Bilateral	Delayed	Hemispheric cyst	No
ID-202	Male 3.8 y	No		Multifocal S-W	Unilateral	Delayed	No	Bicuspid aortic valve Nystagmus

*S-W* Spikes-Wave

### Subcortical heterotopia (SUBH)

**Fig. 2:**
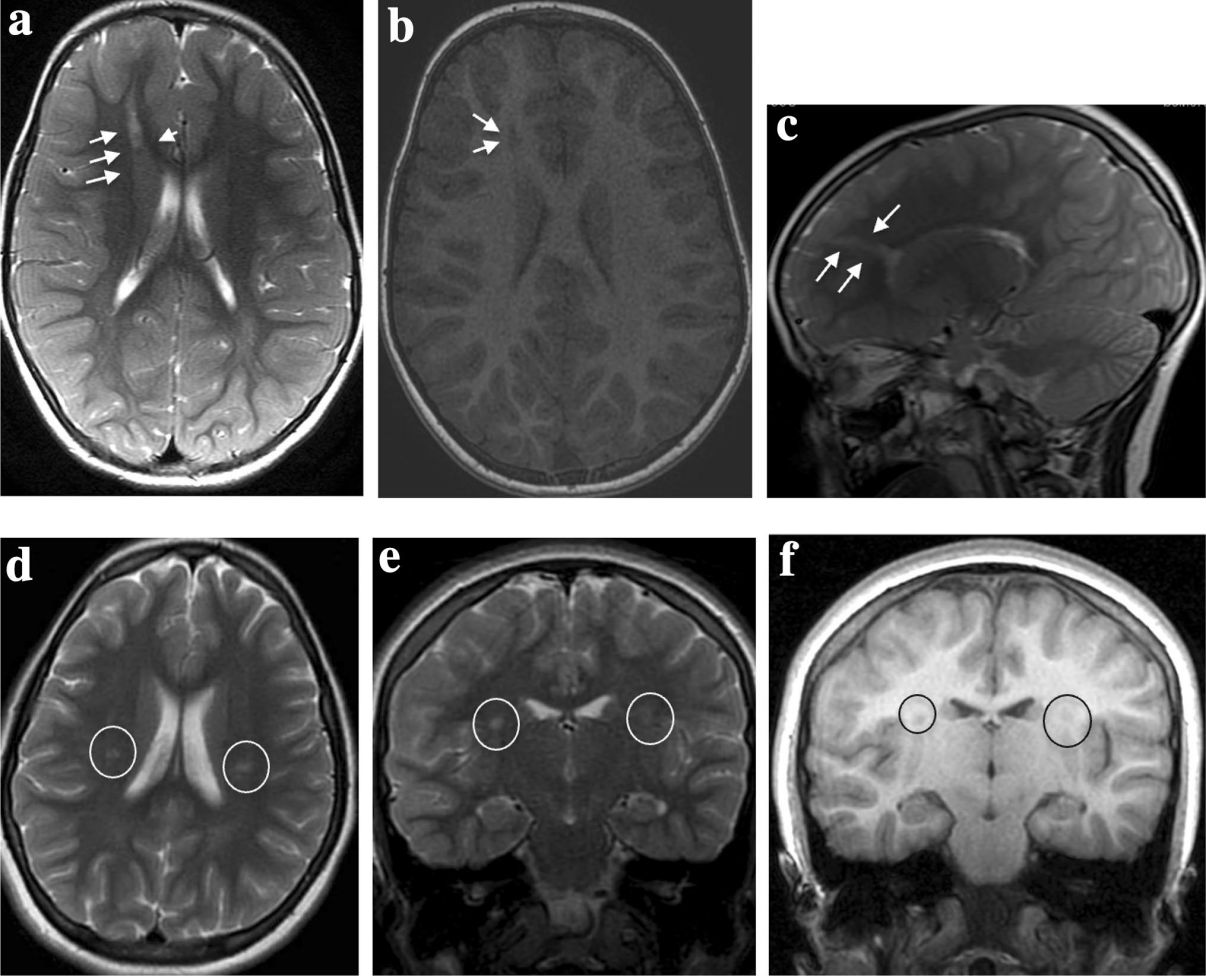
*Subcortical heterotopia (SUBH).* **a**–**c** *(ID 302)* Non-contrast enhancement magnetic resonance imaging, Axial T2- weighted image (**a**) and T1-weighted image (**b**) and mid-sagittal T2-weighted image show mantle stripe, isointense to the cerebral cortex with a serpiginous pattern, which stretches in oblique cranio-caudal direction from the right frontal cortico-subcortical region to the anterior horn of the homolateral lateral ventricle (arrows), compatible with sub-cortical heterotopia. **d**–**f** *(ID 303)* Non-contrast enhancement magnetic resonance imaging axial **d** and coronal **e** T2-weighted image and coronal T1-weighted image **f** shows a bilateral nodule of SNH (circles), isointense to gray matter

This group includes three children, all males, with mean age of establishing a diagnosis of 9 years (± 1.8). Table 3 shows the clinical features. Figure 2 shows the main characteristics and intensity of periventricular nodules on MRI.

**Table 3:** Data of three probands with subcortical heterotopia (SUBH)

ID patient	Patient sex and age at the diagnosis	Seizure	Seizure tupes types	EEG	Heterotopic Localisation	Psychomotor development	Other CNS manlformations	Systemic malformations
ID-301	Male 8.5 y	Yes	FS	F. S-W Right	Nodular	Normal	No	Strabismus
ID-302	Male 7.5 y	Yes	F to Bil	F to Bil	Nodular	Normal	No	No
ID-303	Male 11 y	Yes	FS	Normal	Nodular	Normal	No	No

*FS* focal seizures, *F to Bil* focal to bilateral tonic–clonic seizures, *F. S-W* frontal spike and wave frontal

## Discussion

In this retrospective review, we selected 22 children affected by HET: 17 presenting with PVNH 2 with SBH, and 3 with SUBH. HET manifests with variable clinical expression, mainly presenting with DD/ID, epilepsy and other brain and systemic malformations [3, 9–12]. In this series, 12 out of 22 (54%) subjects showed delayed cognitive function, and 11 out of 22 subjects (50%) showed epilepsy with a lower frequency than the results from other studies that reported cognitive disability and epilepsy in a greater number of cases [11, 12].

In literature HET is associated with epilepsy [13–15]; about this, in our experience 11/22 patients (50%) developed epilepsy. Several studies reported that periventricular heterotopia is the type of GMH more associated with the epilepsy (80–90%) [12, 16], which was more common focal seizure than general [12, 13], and more detected in females than males [4, 15]. Differently, in our periventricular group we detected epilepsy in 8/17 patients (47%), with a female predominance (female to male ratio 5:3). About the type of epilepsy, we found generalized seizures in 4/8 patients, followed by two patients with infantile spasms and two with focal seizure. In the report of Srour et al., all patients with infantile spasms showed bilateral lesions [16]: in our case series, only 1/2 with infantile spasms showed bilateral lesions in accordance with the study of Hung et al. [11]. Consistent with the literature, the most common EEG abnormality was focal epileptiform.

About the subcortical group, we collected three patients affected by epilepsy, two focal type and one generalized seizures, and no other anomalies. Only one patient had strabismus. Raza et al. report a study with ten patients affected by subcortical heterotopia [17]: they describe an association with subcortical heterotopia, central nervous system (CNS) anomalies and neurological dysfunction. Probably due to the low number of patients, we did not detect these findings.

About the band group, we collected two patients with psychomotor delay, without seizures. One of the two patients had hemispheric cystic. Hung et al. describe 10 patients with band heterotopia [11], associating it with brain anomalies and congenital malformations. As the precedent group, due to the low number of patients, it is difficult compare our results.

**Fig. 3:**
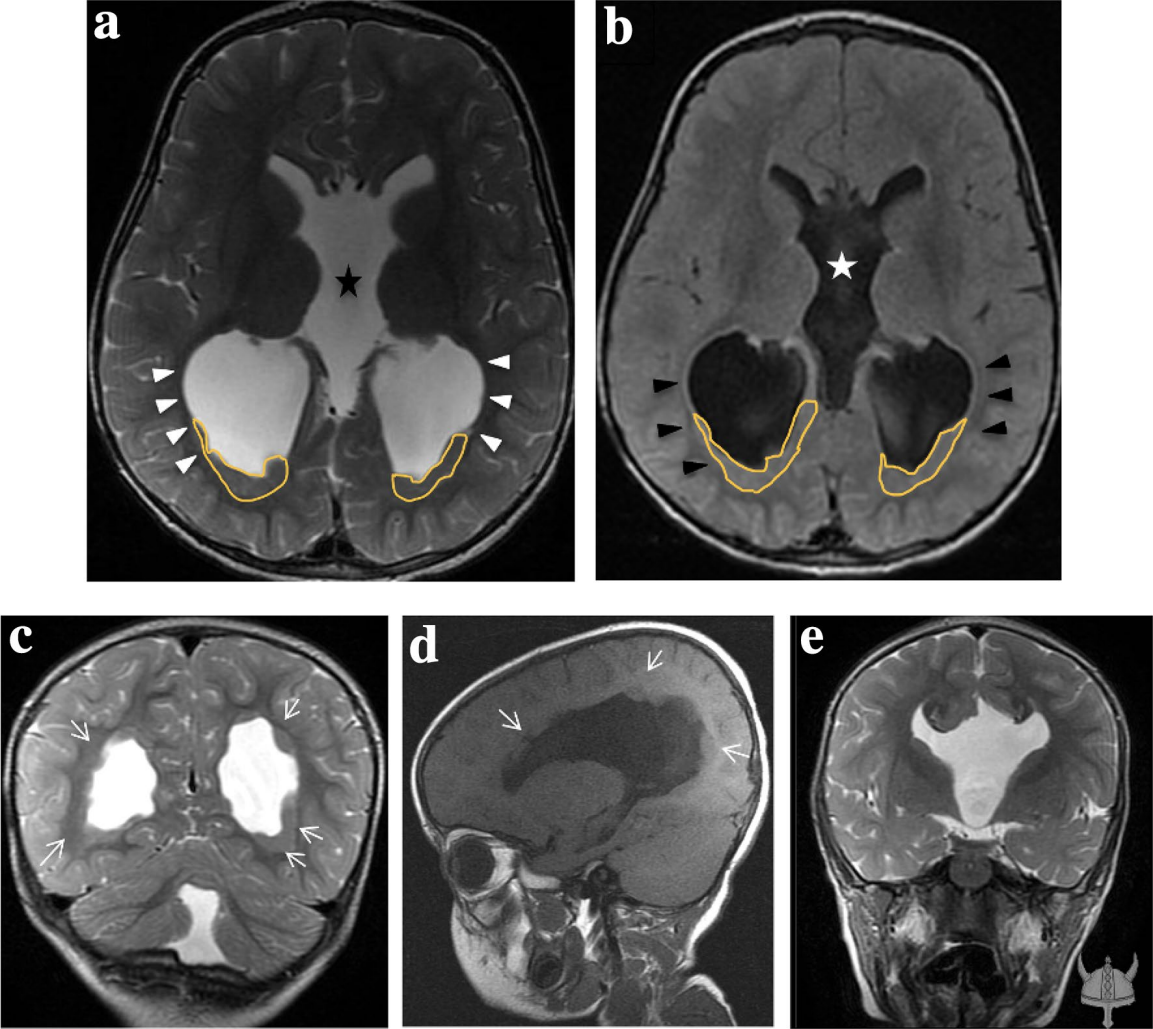
*(ID 108): Agenesis of the corpus callosum and cavum septum pellicidum, ventriculomegaly and cerebellar vermis atrophy in patient with bilateral periventricular nodular heterotopia (PVNH)*. **a**, **b** Non-contrast enhancement axial T2-weighted MR image **a** and T1-weighted MR image **b** shows enlargement of the ventricles in particular the images show dilatation of the third ventricle (star) and lateral ventricles with widely spaced parallel bodies (arrowheads) in patient with bilateral periventricular nodular heterotopia (white lines). **c** Unenhanced T2-weighted MR coronal image show bilateral periventricular nodular heterotopia (PVNH) (arrows), enlarged lateral ventricles and cerebellar vermis atrophy. **d** Unenhanced T1-weighted MR midsagittal image show dysmorphic ventricular system and periventricular nodular heterotopia (arrows). **e** T2 -weighted MR coronal image shows the “Viking helmet appearance” refers to the morphology of the lateral ventricles in the coronal plane in patients with dysgenesis of the corpus callosum. The cingulate gyrus is everted into narrowed and elongated frontal horns

**Fig. 4:**
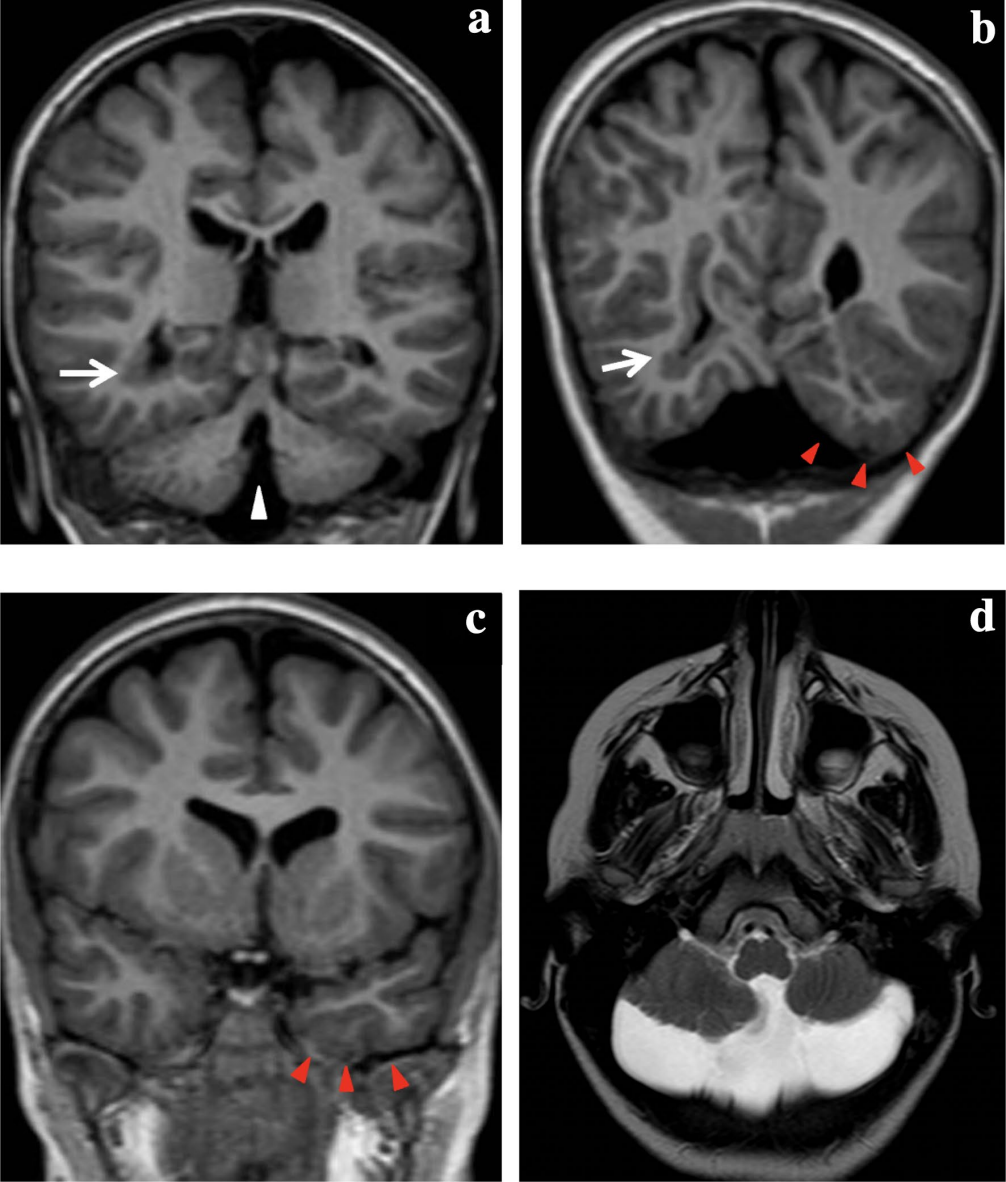
*(ID 103) Brain malformations associated with monolateral periventricular heterotopia (PVNH)* **a**, **b** T1-weighted coronal MR images demonstrates nodular periventricular heterotopia (white arrows) in the right lateral ventricle, cerebellar vermis hypoplasia **a** (white arrowhead) **b**, **c** T1-weighted coronal MR images shows pachygyric appearance of the left parahippocampal gyrus with a ‘nubby’ appearance to the cortical surface (white arrows) **d** T2-weighted assial MR image show cerebellar hemisphere and vermis hypoplasia

**Fig. 5:**
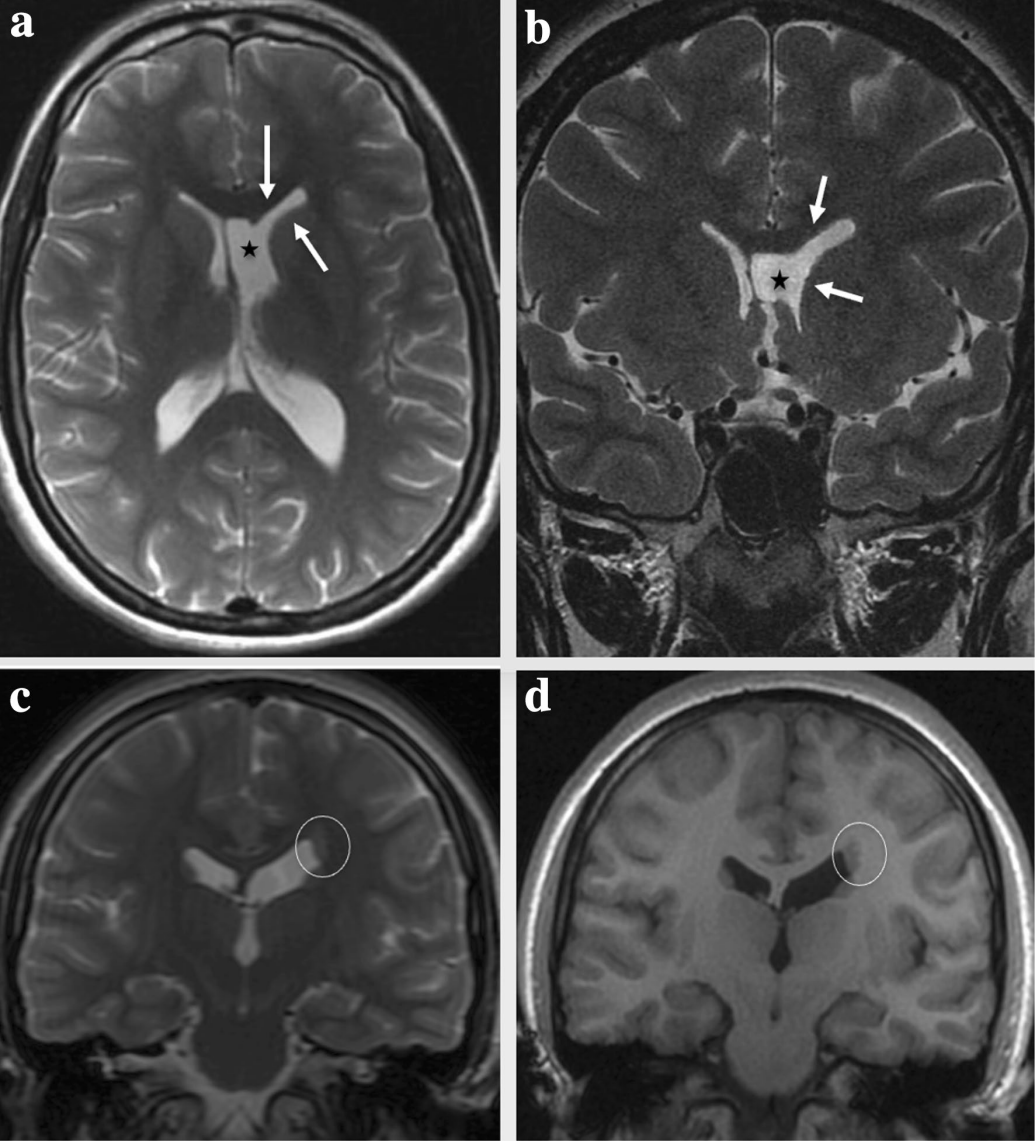
*(ID 109): Dysmorphic left lateral ventricles and periventricular nodular heterotopia (PVNH)*. **a**, **b** Unenhanced axial T2-weighted (T2w) magnetic resonance image **a** and unenhanced coronal T2-weighted (T2w) magnetic resonance image **b** shows dysmorphic left lateral ventricles (white arrows) that appear fused with cyst of the cavum septum pellucidum (black star). **c**, **d** Coronal T2-weighted (T2w) magnetic resonance image **c** and T1 3D FSPGR (fast spoiled gradient echo) coronal magnetic resonance image **d** shows periventricular nodular heterotopia (white circle)

HET can be associated with other CNS anomalies: in our case series, especially in periventricular group, we noted the recurrence of ventriculomegaly (8/17) in accordance with the literature (Fig. 3). In contrast to the paper Hung et al. and Srour et al. [11, 16], we found that bilateral heterotopia are more likely have ventriculomegaly instead of unilateral type. Ventriculomegaly is followed by agenesis of corpus callosum (3/17), cerebellar vermis hypoplasia (Fig. 4a) (2/17) and polymicrogyria (2/17) (Fig. 4b, c). In addition, we found in 2/17 hemispheric cyst (Fig. 5a, b).

In accordance with the data of Hung et al., we report the case of a patient with Dandy-Walker cyst and the case of a patient with Ehlers-Danlos syndrome. Advances in genetic show a correlation between connective tissue disorder and periventricular heterotopia [18]: our experience confirms this date.

Interestingly, we report the case of a patient with periventricular heterotopia and Aicardi syndrome, showing seizures and agenesis of corpus callosum, typical features described in the syndrome [19].

**Fig. 6:**
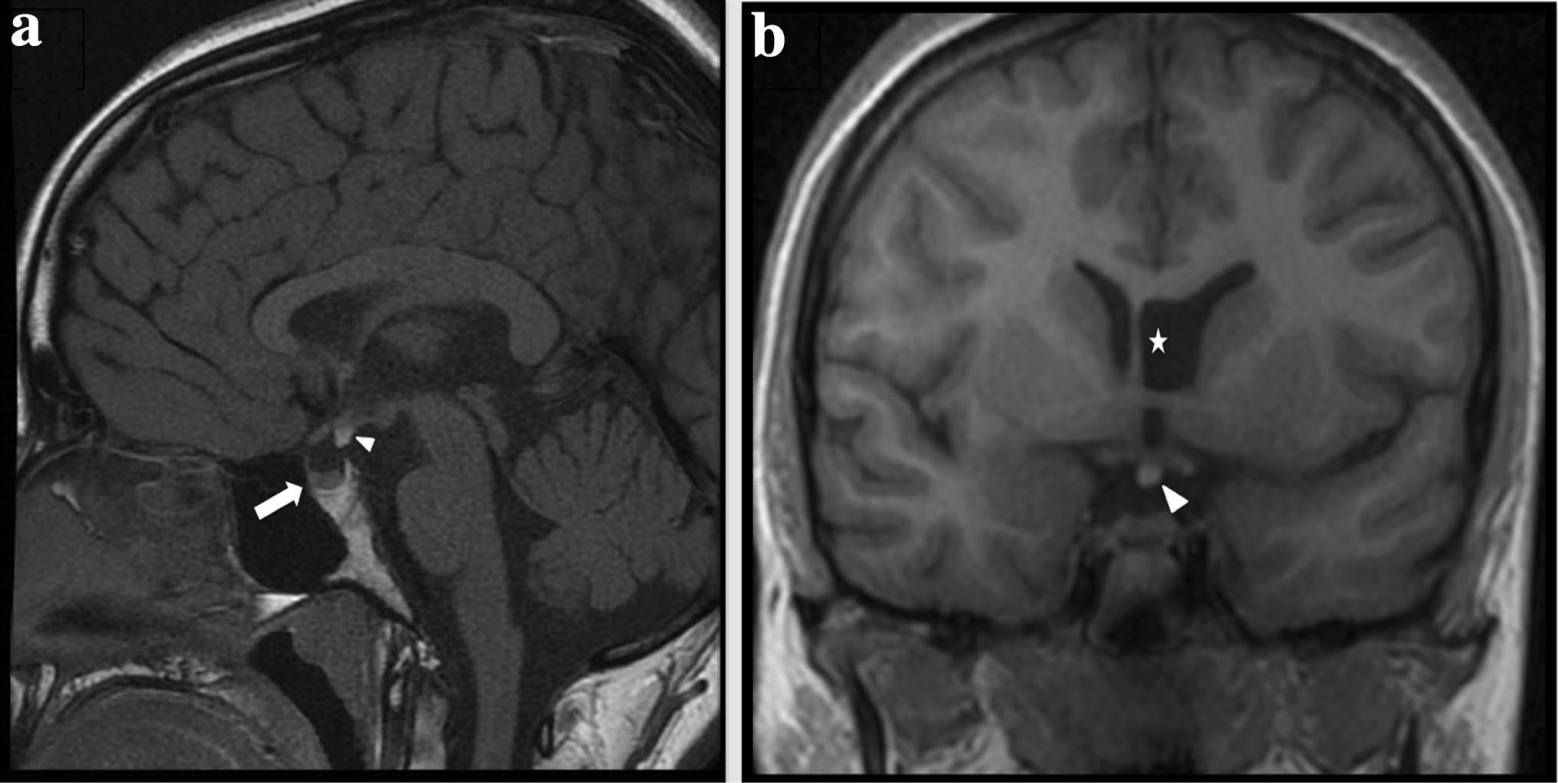
*(ID 109): Ectopic posterior pituitary lobe*. **a**, **b** Unenhanced T1-weighted (T1w) midsagittal and coronal magnetic resonance images shows hyperintensity at the median eminence, which corresponds to ectopic posterior pituitary lobe (arrowheads). Anterior pituitary lobe appears normally localized although small and flattened (arrow). **b** Dysmorphic left lateral ventricles that appear fused with cyst of the cavum septum pellucidum (white star)

About the other systemic malformations, our experience shows a correlation between heterotopia and cardiac diseases, previously described by other studies [11, 16]. Also, we found ocular abnormalities, especially strabismus, in 6/22 patients. Interestingly, we found two patients with growth hormone deficiency (GH) and heterotopia. We attribute this incidence in our case series, never described before, because our center is a reference department of endocrinology and neurology pediatric. In these patients MRI did not show pituitary lesions, so they received diagnosis of GH deficiency idiopathic. In literature, growth hormone deficiency and heterotopia are rarely correlated, such as Vilboux et al. in a Joubert patient [20]. Mitchell et al. reported a study with a possible correlation between periventricular heterotopia, ectopic posterior pituitary lobe and GH deficiency [21]. In our case series, one patient had ectopic posterior pituitary lobe and periventricular heterotopia (Fig. 6); differently, in two patients with GH deficiency MRI did not show anomalies to the posterior pituitary.

Our study had some limitations. The study population was small, especially for the subcortical and band group. All the data were obtained retrospectively. Our patients were only from a single tertiary hospital in Catania. In addition, genetic testing was not performed in our cohort. Our report confirms that heterotopia may be a clue of different and various cerebral and systemic anomalies; for this reason, in case of GMHs for the clinicians it’s important value the cardiac function, ocular abnormalities and eventually skeletal or renal anomalies.

## Declarations

### Data availability

The data used to support the findings of this study may be released upon application to the corresponding author, who can be contacted at alessandradinora@gmail.com.
